# Partial and directional relationships between negative metacognitive beliefs and the big five personality traits

**DOI:** 10.3389/fpsyt.2026.1897206

**Published:** 2026-09-11

**Authors:** Henrik Nordahl, Frederick Anyan, Odin Hjemdal, Adrian Wells

**Affiliations:** 1 Department of Psychology, Norwegian University of Science and Technology, Trondheim, Norway; 2 School of Psychological Sciences, Faculty of Biology, Medicine and Health, The University of Manchester, Manchester, United Kingdom; 3 Research and Innovation, Greater Manchester Mental Health NHS Foundations Trust, Manchester, Manchester, United Kingdom

**Keywords:** big-5, DAG, metacognitive beliefs, personality, S-REF, traits

## Abstract

**Introduction:**

Personality traits represent consistent patterns of expressions in an individual’s cognition, behaviour and emotion and are therefore relevant to psychopathology and present markers for psychological vulnerability. The S-REF model has implications for personality and trait theory because it describes higher-order cognitions involved in self-regulation, persistence and the stability of cognitive-behavioural patterns. Variability in features of the S-REF should be associated with personality dimensions and most strongly associated with those reflecting stable psychological vulnerability. Individual differences in metacognitive knowledge representing beliefs about the personal uncontrollability and dangerousness of thoughts is a central variable in the S-REF. The present study aimed to test the hypothesis that such negative metacognitions are associated with personality factors, with the most robust relationships predicted to be with personality dimensions representing psychological vulnerability (e.g., neuroticism). However, we also predicted wider (but weaker) associations between the MCQ factor and a range of personality dimensions because negative metacognitive beliefs in the S-REF model influence the stability of broader cognition-behaviour patterns.

**Methods:**

We tested relationships among 296 individuals who completed the full NEO-PI-R. Using a network approach, the structure of partial correlations between negative metacognitions and Big Five personality traits were visualized and explored with an exploratory directed acyclic graph (DAG) of the conditional-dependence structure among these variables.

**Results:**

Negative metacognitions showed the highest expected influence in the network. The conditional-independence pattern was more consistent with negative metacognitive beliefs preceding neuroticism than the reverse, but this ordering should not be interpreted as evidence of a causal relationship due to the cross-sectional design.

**Conclusion:**

The results are consistent with predictions based on the S-REF model, that negative metacognitive beliefs are associated with, and structurally central among personality dimensions linked to psychological vulnerability. The directional findings are offered as an exploratory hypothesis for future longitudinal or experimental testing rather than as established causal or mechanistic relationships.

## Introduction

Trait models of personality have been a dominant paradigm in psychopathology literature and define personality as individual differences in cognition, affect, and behaviour that are relatively stable across time and situations. The Five Factor Model (FFM) has been particularly influential and received substantial empirical support (e.g., 1). Traits identified at the highest level in this model include neuroticism, extraversion, openness, agreeableness, and conscientiousness, and they are commonly referred to as the big five (e.g., 2).

The FFM has made important contributions to theories and research approaches in the study of psychopathology. For example, neuroticism, extraversion, and conscientiousness reliably correlate with emotional disorders (3), maladaptive variants of the big five traits define personality disorder syndromes in the most recent diagnostic manuals (e.g., 4), and the big five correlate with psychological vulnerability and resilience (5). Among the big five, particularly (high) neuroticism is considered a marker for psychological vulnerability and disorder (e.g., 6–8) with smaller and additional contributions from other traits (e.g., 9).

Some have argued that personality traits linked to psychological vulnerability and disorder should be targeted in psychopathology interventions (10, 11) while others are more critical to this approach as the traits do not bring more specific information about etiological mechanisms (e.g., 12). Simplistic conclusions regarding the nature of personality traits and vulnerability/disorder associations are unlikely to advance the field, especially if they are not linked to mechanisms of self-regulation. It is likely that there are common underlying psychological processes that can account for the personality traits and psychopathology interrelation (e.g., 13) that are more suitable for being conceptualized and targeted in interventions.

The Self-regulatory Executive Function (S-REF) model (14–16), formulates a higher-order *metacognitive control system* (MCS; 16) which contributes to consistency and repetition in behaviours, cognition and emotion, in the context of failures in self-regulation. Processing configurations within this system, which draw on dysfunctional metacognitive beliefs (e.g., worrying is uncontrollable; thoughts are important), are a central to psychological vulnerability. When such configurations are activated, they cause a *cognitive attentional syndrome* (CAS) which consists of negative thinking styles (e.g., worry and rumination), threat monitoring (i.e., strategic attention directed toward perceived threats), and maladaptive coping strategies (e.g., avoidance). Of particular importance are negative metacognitive beliefs which concern the uncontrollability and dangers of cognition, as they contribute to reduced investment in adaptive mental regulation strategies when exposed to stressors and self-regulatory discrepancies leading to persistent run-away CAS loops (17). Thus, the S-REF and MCS model, hence forward referred to as the metacognitive model, predicts that negative metacognitive beliefs should be associated with personality traits, and most strongly those traits associated with psychological vulnerability and disorder (e.g., neuroticism). However, relationships with wider personality traits are also likely to be present because of the consistency and stability of personality patterns, and the model views consistency as partly a function of metacognition.

In line with the metacognitive model, negative metacognitive beliefs show a significant positive and moderate association with neuroticism in several studies (e.g., 18–22). Furthermore, significant negative associations of small strength have been reported with extraversion, agreeableness, and conscientiousness, while negative metacognitive beliefs also appear to correlate with openness (e.g., 20–22). However, it should be noted that these studies have used brief scales to assess personality traits. Moreover, and to the best of our knowledge, no study has yet tested the partial or statistically directional relationships between negative metacognitive beliefs and big five personality traits (i.e., controlling for the association between all other variables) and therefore the present study is an important step towards elucidating the unique relationships and hierarchical structure between these variables.

We applied a network modelling approach to explore the association between negative metacognitive beliefs and big five personality traits using state of the art indicators (i.e., the MCQ-30 uncontrollability-and-danger subscale and full NEO-PI-R). We computed a network model of negative metacognitive beliefs and big five personality traits to describe and visualize pairwise connections among them. We were especially interested in the computation of centrality metrics that describe the most structurally prominent nodes in the network structure. We sought to empirically determine which nodes in the network are more central to the network structure, and relatively more important by computing the expected influence (EI), and to determine the shared variance of each variable in node predictability. As part of the analyses, we verified the robustness and stability of the network structure. However, network models resulting from undirected edges or partial correlations do not inform about the order or direction of association between variables. To explore this further, we applied a Bayesian hill-climbing method to estimate a directed acyclic graph (DAG), which is a directed network whereby each edge has an arrow tip on one end, signifying the direction of probabilistic dependence (23–25). We treat the DAG as exploratory since it describes one directed ordering of the conditional-independence relations among variables that is consistent with the observed, cross-sectional, contemporaneous self-report data. However, it does not by itself establish causal relations since this additionally requires causal sufficiency, correct model specification, and absence of unmeasured confounding, none of which can be verified with the present cross-sectional design (23, 26). The DAG analysis can indicate whether the presence of node B (“downstream”) is probabilistically consistent with the presence of node A (“upstream”) more than the reverse, within the assumptions of the model.

Based on the metacognitive model (14, 16), our specific hypotheses were as follows:

- Negative metacognitive beliefs will show a unique relationship with neuroticism. Additionally, we were interested to explore any independent associations with other personality traits because metacognition is implicated in cognitive stability more widely and not just in emotional vulnerability traits.
- Negative metacognitive beliefs will show high expected influence in the network; and
- Negative metacognitive beliefs will occupy an “upstream” position in the exploratory DAG ordering, consistent with the idea that negative metacognitions may contribute to personality traits.

Importantly, we note that recovering hypothesized variable ordering, *if observed*, would be consistent with an assumption already built into the S-REF model rather than an independent test of it, since the model itself specifies this direction of influence. The reverse direction, or a shared common cause, cannot be ruled out by the present cross-sectional design, but would not be consistent with the mechanistic position of the S-REF model (14).

## Methods

### Participants and procedure

The current study is based on an anonymous self-report survey completed with pen and paper as part of an introduction course to personality psychology at a Norwegian university. Information about the survey and invitation to participate was provided as part of a lecture on normative personality traits. Participation was voluntary, and there were no course-credit arrangements. To be eligible, participants had to be able to read Norwegian and consent to take part in the study. Safeguards for voluntariness included clear information about the survey, ensuring that participation was voluntary, offering time to consider the decision about participation, and not providing any rewards to take part. One possible incentive to take part, which was not explicitly stated when informing about the survey, was to get first-hand experience with completing the NEO-PI-R. We did not collect specific data on response rates, but around 350 students registered for the course (but was not necessarily present at the specific date of recruitment). The sample consisted of 296 (77.7%) females, and the mean age was 22.34 (*SD* = 3.29) years old. Hundred and ninety-nine (67.2%) were in their first year at the university.

### Measures

The Revised Neuroticism, Extraversion, Openness – Personality Inventory (NEO-PI-R; 27) is a 240 item self-report scale assessing the big five traits and their corresponding six lower-order facets. Each item is scored on a scale ranging from 0 (“strongly disagree”) to 4 (“strongly agree). The NEO-PI-R has been validated in Norway (28) and we performed normative transformation based on Norwegian norms for sex and age. In the current study, the internal consistency of the big five was adequate to good (Cronbach alphas; N = 0.83, E = 0.81, O = 0.75, A = 0.74, C = 0.82).

The metacognitions questionnaire 30 (MCQ-30; 29) was used to assess negative metacognitive beliefs (e.g., “worrying is uncontrollable”) in the S-REF model. The MCQ-30 is a 30-item self-report scale assessing five subscales with 6 items. Each item is scored on a scale ranging from 1 (“do not agree”) to 4 (“agree very much”), so that subscales range from 6 to 24 where a higher score indicate stronger endorsement of the belief domain in question. In the current study, the internal consistency of the subscale assessing negative metacognitive beliefs was good (*α* = 0.83).

### Statistical analyses

All analyses were performed in R-studio statistical software (30). Software package versions used in this study were *R 4.4.2; networktools 1.6.0, bootnet 1.8, qgraph 1.9.8, mgm 1.2-15, bnlearn 5.1, dplyr 1.2.0, huge 1.5, corrplot 0.95, psych 2.6.1, corpcor 1.6.10, ppcor 1.1, MVN 6.3.* All supporting materials for reproducible analyses and codes are available on the Open Science Framework platform at https://osf.io/zr2se/.

#### Data preparation

Following psychological network literature guidelines (31), we applied nonparanormal transformations using *huge* package (32). Next, we verified that the correlation matrices submitted for analyses were positive definite since violation will mean that the variables in the network structure may be linear combinations with themselves. We used the Hittner method via the *goldbricker* function to search for highly intercorrelated variables (*r* >.50). All 296 participants had complete data on the six analytic variables, so no observations were excluded for missingness. The sample derives from an existing dataset collected for a related purpose rather than from an *a priori* power analysis for network estimation, which we note as a limitation.

#### Network structure

Network components (i.e., nodes) were six variables including: *negative metacognitive beliefs, neuroticism, extraversion, openness, agreeableness and conscientiousness.* The associations between nodes are referred to as edges (31). Statistically, edges represent partial correlations between two variables, controlling for all the other variables or nodes (31). We computed correlation matrices of the variables as input for a Gaussian Graphical Model (GGM) that estimates regularized pairwise association between all variables (31), thereby removing spurious and trivial, potentially ‘false positive’ edges. The network structure was estimated using *qgraph* (33), which implements the graphical LASSO (Least Absolute Shrinkage and Selection Operator) regularization in combination with Extended Bayesian Information Criterion (EBIC) model selection (gamma = 0.5; *nlambda* = 100, *lambda.min.ratio* = 0.01, refit = FALSE, i.e., *qgraph::EBICglasso* defaults otherwise). Correlations were estimated on the nonparanormal-transformed data via *qgraph::cor_auto* (no ordinal variables were present). Given the low dimensionality of this six-node network (15 possible edges), regularization may introduce bias without a clear inferential advantage. We therefore also report the complete zero-order and unregularized partial-correlation matrices with 95% confidence intervals, and an unregularized GGM sensitivity analysis. We used nonparametric bootstrapping with replacement for the confidence regions of the edge weights to test the stability of the edges in the network structure as well as the difference between edge weights via the *bootnet* package (31).

To determine the importance of nodes/variables in the network structures we compute the expected influence (EI) centrality (34), and the node predictability, which indicates the shared variance of a given node with its surrounding nodes in the network using the *mgm* package (35). The one-step EI was calculated to quantify which node/variable had the most relative importance in the network structure. The EI provides a more accurate estimate of node centrality compared to strength centrality values, which only takes the absolute values of edges into account while the EI accounts for both positive and negative edge values when the network structure contains positive and negative edges. Higher expected influence values indicate greater centrality and thus greater importance in the network structure (34). A person-dropping bootstrap procedure was applied to verify the stability of the expected influence, and a bootstrap expected difference test was applied to determine significant difference between the nodes in terms of the expected difference (31, 34).

#### Directed acyclic graph

Following a recent study (36), we estimated a DAG with the Bayesian hill-climbing algorithm using the *bnlearn* package (25). In a DAG, conditional-probability relations among variables are represented as a directed graph. We treat this as an exploratory representation of one ordering of the conditional-independence structure rather than as an established causal model, since causal interpretation additionally requires causal sufficiency, correct model specification, and absence of unmeasured confounding, none of which can be verified from the present cross-sectional, contemporaneous self-report data (24, 37). To clarify which arc directions are identifiable from the observed conditional independencies, we additionally computed the completed partial DAG (CPDAG) representing the Markov equivalence class of the learned network, and report which arcs are identifiable versus not identifiable from the data.

Using a bootstrap function, this approach learns the features of the model through iteratively adding and removing different candidate edges and reversing their direction until reaching an optimal goodness-of-fit target score (i.e., Bayesian Information Criterion – BIC). Then, various edges connecting various node pairs are estimated through 50 random starts with 100 perturbations by several attempts to connect, remove and reverse edges, thus avoiding local maxima problems to return a model with an optimal BIC value (36, 37). We additionally checked sensitivity of the learned structure to the random seed and to fitting on the nonparanormal-transformed scores used for the GGM.

The stability of the DAG was determined by recommended guidelines (37), applied in recent studies (36). We bootstrapped 10,000 samples and then averaged the resulting 10,000 bootstrapped networks to compute a final model via two steps. Step one verifies frequency of edges in the 10,000 bootstraps and then an optimal recommended cut-point (38) was applied to retain edges, thus resulting in a model with high sensitivity and specificity (36). In step two, a minimum edge direction (i.e., % of fitted networks in which a given edge has a given direction) of 50% in the surviving bootstrapped network was reported in the final model. The final DAG, therefore, resulted in a model with directional arrows that are present based on: i) an optimal cut-point, and ii) whose direction of connection featured in the predicted, potentially ‘causal’ direction in more than half of the fitted models from the bootstrap procedure. The optimal bootstrap inclusion threshold for this sample was 0.4988 (i.e., arcs present in more than 49.88% of bootstrapped networks were retained). The bootstrap arc strength (frequency of the arc’s presence in either direction) is reported alongside directional probability for every retained arc in **Table 1**, and for all possible directed arcs.

**Table 1:** Arcs retained in the exploratory directed acyclic graph, with bootstrap arc strength, change in BIC, and directional probability.

Arc (from → to) in the exploratory DAG	Value determining arrow thickness		
	BIC	Directional probability	Arc strength
Negative beliefs about the uncontrollability and corresponding danger of worry → Neuroticism	-81.11	0.84	1.00
Extraversion → Openness	-22.01	0.50	1.00
Conscientiousness → Neuroticism	-17.26	0.84	0.99
Neuroticism → Extraversion	-9.97	0.86	0.81
Agreeableness → Neuroticism	-1.15	0.71	0.55

BIC = Change in Bayesian Information Criterion when the arc is removed from the DAG. Larger absolute values indicate arcs more strongly supported within this exploratory model, not causally confirmed effects. Arcs shown were retained because their bootstrap arc strength exceeded the optimal inclusion threshold of 0.4988 (38. Complete bootstrap arc-strength values for all possible arcs, and the completed partial DAG (CPDAG) indicating which arc directions are identifiable from the data, are reported in the **Supplementary Material**. Directional probability values represent the proportion of the 10,000 bootstrapped networks in which the arc was present in the reported direction. The extraversion → openness arc’s directional probability of 0.50 is uninformative and should not be interpreted as favouring either direction.

## Results

**Figure 1:**
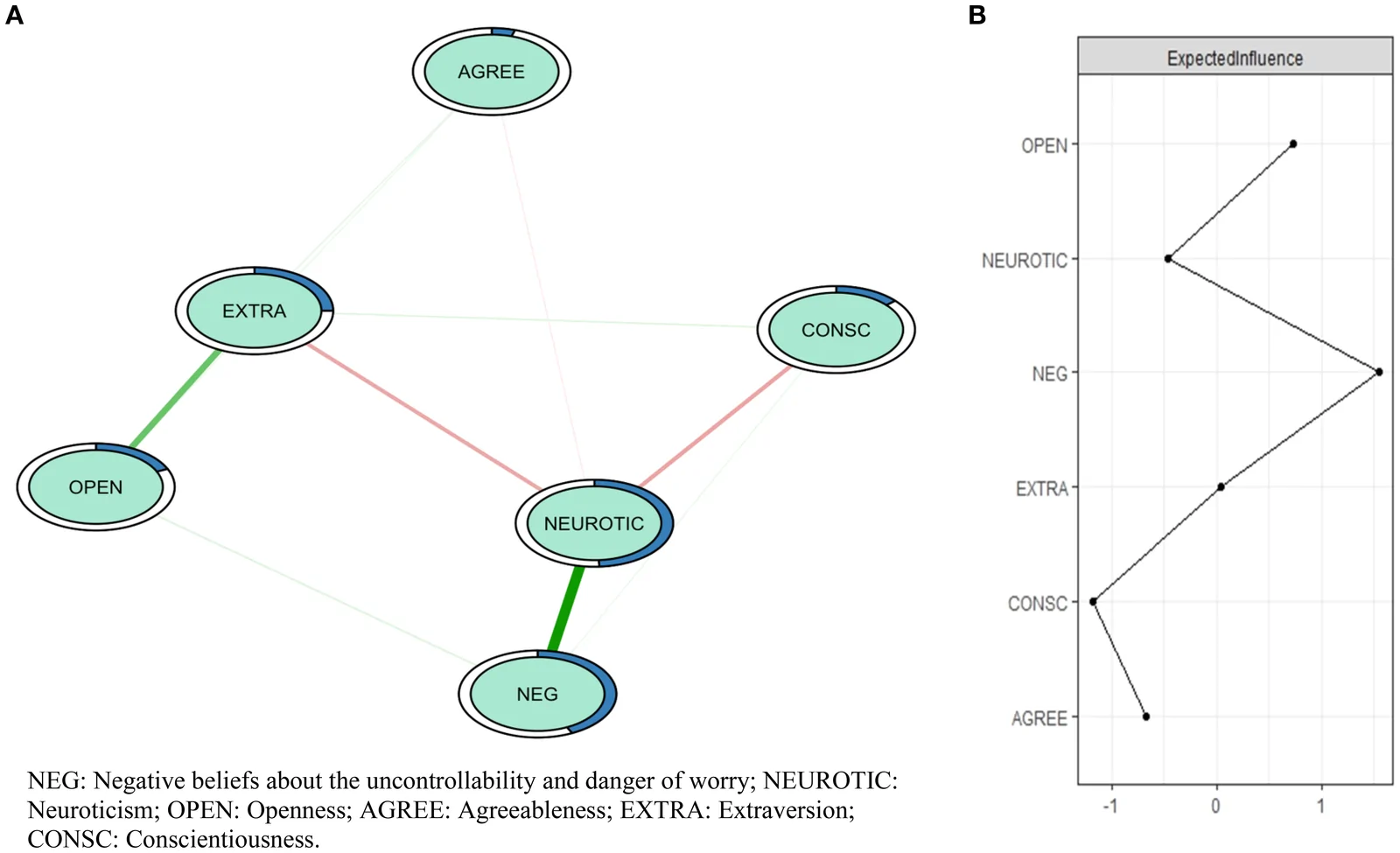
**(A)** Regularized partial correlation network structure showing connections between negative metacognitive beliefs and dimensions of personality traits. Green lines represent positive partial correlations, and red lines represent negative partial correlations while the thickness indicates strength of connection. The rings around the nodes indicate the proportion of explained variance in that node by all other nodes. **(B)** Expected influence values for network nodes. Higher values indicate greater expected influence. NEG, Negative beliefs about the uncontrollability and danger of worry; NEUROTIC, Neuroticism; OPEN, Openness; AGREE, Agreeableness; EXTRA, Extraversion; CONSC, Conscientiousness.

Descriptive statistics, the zero-order correlation matrix (with 95% confidence intervals), and normality tests are contained in the **Supplementary Material**, page 2. The regularized partial-correlation (adjacency) matrix used as input to the network in **Figure 1A** is reported separately from the zero-order matrix, and an unregularized partial-correlation matrix is provided as a sensitivity check (**Supplementary Material**, page 3). The zero-order correlation matrix was verified to be positive definite and no redundant variables with high intercorrelations were found (goldbricker function, Hittner method, *r* >.50). However, the data was found to be nonnormally distributed, Mardia’s multivariate skewness, *MS* = 182.311, *p* <.001; Mardia’s multivariate kurtosis, *MK* = 4.356, *p* <.001. Therefore, we applied a nonparanormal transformation following guidelines in the psychological network literature, prior to estimating the GGM.

### Network structure of negative metacognitive beliefs and personality traits

**Figure 1A** display a visualization of the network model. Node placement was determined by Fruchterman and Reingold (39) algorithm whereby nodes nearer to the centre of the graph tend to have the strongest connections with other nodes. A thicker edge denotes a larger association. Green edges represent positive regularized partial correlations, whereas red ones represent regularized negative partial correlations. *Negative metacognitive beliefs* and *neuroticism* were the most centrally placed variables as they shared the biggest edge weight (.55), followed by *openness* and *extraversion* whose edge weight was (.32). Notably, *neuroticism* (*R^2^* = 0.49) followed by *negative metacognitive beliefs* (*R^2^* = 0.43) had the highest shared variance, which means that they shared strong connections with surrounding nodes in the network structure. Edge weights for all connections are contained in the adjacency matrix in the **Supplementary Material**, page 3. **Figure 1B** displays estimates of the expected influence of all variables while other centrality estimates are contained in the **Supplementary Material**, **Supplementary Figure 1** in page 4. **Supplementary Figure 2** in the **Supplementary Material**, page 5, contains a visualization of the unregularized network structure for sensitivity check. The edge patterns, overall, is very similar to the regularized network.

*Negative metacognitive beliefs* showed the highest expected influence (0.67), indicating that it functioned as the primary influential hub in the network. The expected influence is a descriptive function of the signed adjacent edge weights, so it indicates that negative metacognitive beliefs had the largest combined association with the rest of the network. We report it here as a structural characteristic of the estimated network.

Neuroticism, however, had the highest betweenness (7.0). Betweenness describes how often a node lies on the shortest path connecting other node pairs in the estimated network, suggesting a variable’s connections in linking other nodes. Like the expected influence, we report betweenness here as a structural characteristic of the estimated network. *Neuroticism* emerged as a key structural node, characterized by very high betweenness (7.0) and high strength (0.98) but comparatively lower expected influence (0.11), implying that it primarily organized connections among nodes in the network.

The stability analyses of edge weights suggest that most edges were fairly stable and that the strongest edges are significantly larger than most others (**Supplementary Figures 3A, B** in **Supplementary Materials**, page 6). The person-dropping bootstrap procedure for stability analyses of centrality measures and the bootstrap expected difference test results are contained in the Supplementary Material (**Supplementary Figures S4A, B**, page 7). The correlation coefficient in the network for expected influence (CS = 0.44) suggests cautious interpretation of the relative ordering of expected-influence estimates.

### Directed acyclic graphs

**Figure 2:**
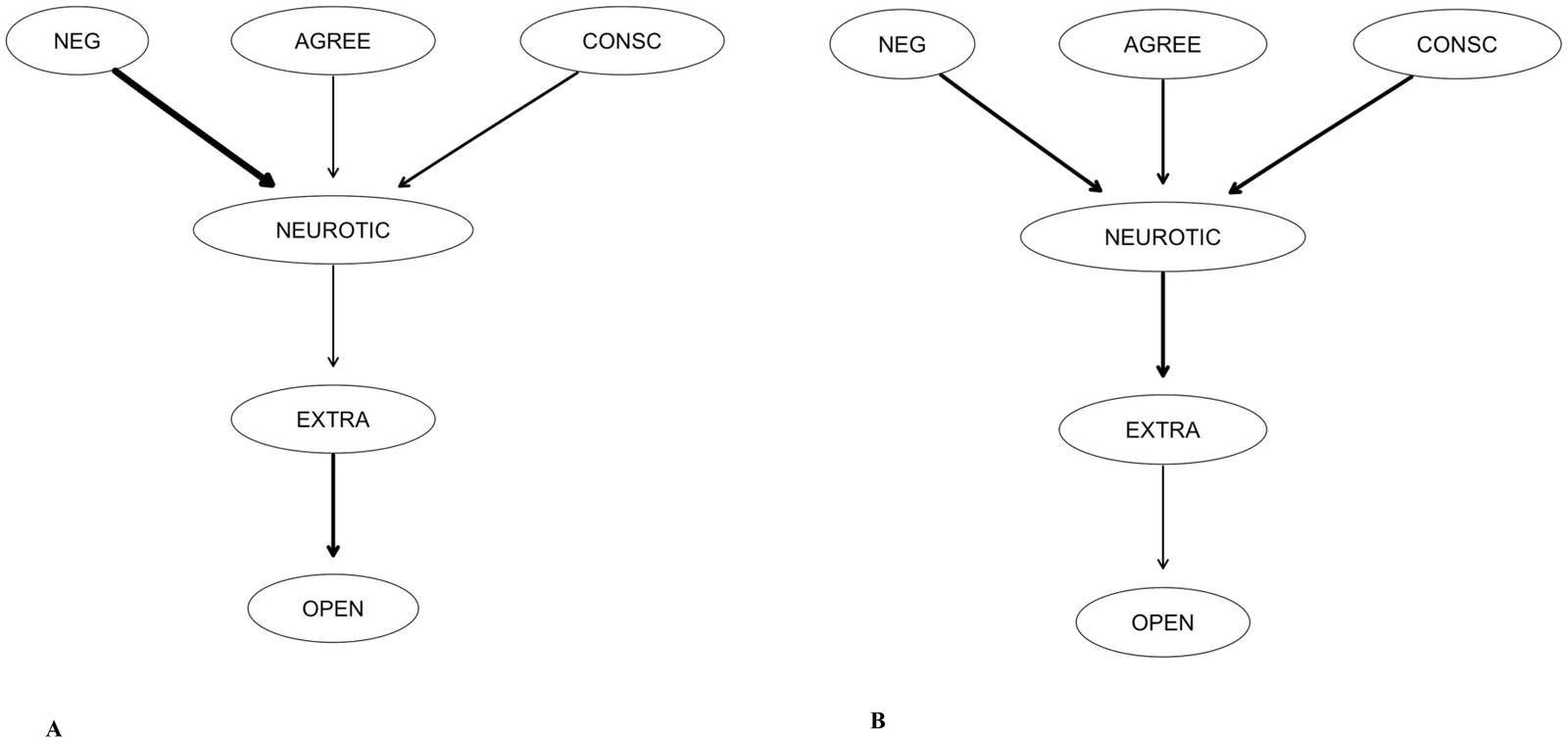
**(A)** Connection strength and arrow importance to model fit in the DAG. Arrow thickness represents their importance to overall mode fit. Thicker arrows show larger contributions to model fit (i.e., Bayesian Information Criterion, BIC) in the potential cause*-and-effect* relations. **(B)** Direction probability in the DAG. Arrow thickness represents directional probability such that thicker arrows reflect greater proportions of bootstrapped networks showing the arrow pointing in the same direction than the reverse direction. NEG, Negative beliefs about the uncontrollability and danger of worry; NEUROTIC, Neuroticism; OPEN, Openness; AGREE, Agreeableness; EXTRA, Extraversion; CONSC, Conscientiousness. DAG, Directed Acyclic Graph.

As stated, the network model does not inform about the order or direction of association between variables. To explore this, we additionally, estimated a DAG, which we present as exploratory throughout the manuscript. The DAG describes one directed ordering of the conditional-independence structure that is consistent with these cross-sectional data, not an established causal model. **Figure 2A** show the DAGs from 10,000 bootstrapped. Arrows in the graph were retained because arrow strength was greater than the optimal cut-point (0.4988; 38). The DAG was estimated on the untransformed scores, and a sensitivity DAG fitted to the nonparanormal- transformed scores used for the GGM recovered exactly the same 6 arcs as the untransformed-score DAG (true positives = 6, false positives = 0, false negatives = 0). In **Supplementary Figure 3A**, arrow thickness represents the change in the Bayesian Information Criterion (BIC; a relative measure of a model’s goodness-of-fit) when that arrow is removed from the DAG. That is, thicker arrows contribute more to model fit and are more strongly supported within this exploratory model. A larger change in BIC indicates a more strongly supported arc, not a causally confirmed effect. Three variables occupied the upstream (source) positions of the exploratory DAG. Based on the arc strength and change-in-BIC values in **Table 1**, *negative metacognitive beliefs, conscientiousness*, and *agreeableness* were positioned upstream of *neuroticism*, with negative metacognitive beliefs contributing the largest change in BIC. In other words, within this exploratory ordering, neuroticism was probabilistically dependent on *negative metacognitive beliefs* more strongly than on *conscientiousness* and *agreeableness*. This is reflected in the change in BIC = -81.11 for the arc from *negative metacognitive beliefs* to *neuroticism*, compared with -17.26 and -1.15 for the arcs from *conscientiousness* and *agreeableness* to *neuroticism*, respectively. In practical terms, within this exploratory model, participants who reported elevated *negative metacognitive beliefs* were also more likely to report elevated *neuroticism* than the converse ordering. We do not interpret this as confirmed evidence that negative metacognitive beliefs trigger, cause, or underlie elevated neuroticism. The design cannot rule out the reverse direction or a shared common cause, and the ordering reflects the best-fitting conditional-independence structure for these data under the model’s assumptions, not a demonstrated causal effect.

Openness was probabilistically dependent on extraversion, which was itself probabilistically dependent on neuroticism, within this exploratory ordering. We note that the *extraversion → openness* arc had a directional probability of exactly 0.50 (**Table 1**), which is a chance level finding, and we therefore do not interpret its direction. It provides no evidence favouring either orientation. We describe the overall pattern as one internally consistent exploratory ordering of the variables’ conditional dependencies, with *negative metacognitive beliefs* positioned earliest, rather than as a chain of causal or cascading events. **Table 1** shows the change in BIC values, bootstrap arc strength, and directional probability for each retained arc in the DAG. The complete bootstrap arc-strength table for all possible arcs is reported in the **Supplementary Material**, page 8. A completed partial DAG (CPDAG) analysis showed all five retained arcs to be compelled (identifiable) in this network’s Markov equivalence class, with no unoriented arcs. Because the *extraversion → openness* arc’s directional probability was exactly 0.50, we do not treat its formal identifiability in the CPDAG as evidence for its specific empirical direction.

In addition to determining genuine arrows that contribute more to model fit in **Supplementary Figure 3A**, we also report the bootstrap-estimated directional probability of each retained arc, i.e., the proportion of the 10,000 bootstrapped networks in which that arc pointed in the reported direction (**Supplementary Figure 3B**). Directional probabilities for the retained arcs ranged from 0.50 to 0.86. The arc *from negative metacognitive beliefs* to *neuroticism* was estimated in the reported direction in 84% of bootstrapped networks, as was the arc from *conscientiousness* to *neuroticism* (84%). The *extraversion → openness* arc’s directional probability was exactly 0.50 and is therefore uninformative about direction, as noted above.

## Discussion

Personality traits such as high neuroticism are strong markers for psychological vulnerability and are positively associated with psychological disorder. However, trait theory faces several conceptual and empirical challenges in elucidating the mechanisms underlying vulnerability and disorder, and it is possible that the correlation between traits and vulnerability/disorder can be accounted for by shared underlying mechanisms. The S-REF model, also known as the metacognitive model (14, 16, 2026) points towards (negative) metacognitive beliefs as central influences on consistency in cognition, affect, and behaviour (i.e., personality traits). In the current study, we used a network modelling approach and assessed the partial and directed relationships between negative metacognitive beliefs and the big five personality traits.

Consistent with our first hypothesis, negative metacognitive beliefs showed a unique positive and strong association with neuroticism, and smaller but unique additional associations with conscientiousness and openness. Negative metacognitive beliefs together with neuroticism were the most centrally placed variables and showed strong connections with surrounding variables. Consistent with our second hypothesis, negative metacognitive beliefs showed the highest expected influence and emerged in a prominent structural position among the six variables in the network. An exploratory DAG was used to describe directed ordering of the conditional-independence structure among the variables. Consistent with our third hypothesis, negative metacognitive beliefs (together with conscientiousness and agreeableness) occupied the upstream positions of this exploratory ordering. Neuroticism was probabilistically dependent on these three upstream variables, of which negative metacognitive beliefs made the strongest contribution to model fit. In other words, negative metacognitive beliefs occupied the earliest position among the variables probabilistically associated with neuroticism in this exploratory ordering. Openness was probabilistically dependent on extraversion which was itself probabilistically dependent on neuroticism, though the extraversion–openness arc’s directional probability (0.50) provides no evidence for either direction.

The results of the current study add to previous research by reporting the unique and directed relationships between negative metacognitive beliefs and the big five personality traits, and by exploring their ordering in a DAG. They are in line with previous studies that have shown a correlational relationship between negative metacognitive beliefs and neuroticism (e.g., 18, 21, 22), and that negative metacognitive beliefs prospectively predict trait-anxiety (19). Previous studies have reported a bivariate negative relationship between negative metacognitive beliefs and extraversion, agreeableness, and conscientiousness, and no relationship with openness (e.g., 20–22). The regularized partial correlation network structure applied in the current study indicated a unique and positive relationship between negative metacognitive beliefs and openness and conscientiousness. We note that with a small set of only moderately intercorrelated nodes, partial correlations for the dominant bivariate association (negative metacognitive beliefs–neuroticism) can be expected to remain close to the zero-order association (.55 versus.63), so this finding should be read alongside the bivariate literature. The exploratory DAG positioned negative metacognitive beliefs, agreeableness, and conscientiousness earliest in the ordering, with negative metacognitive beliefs contributing most to model fit for the arc into neuroticism, and extraversion and openness positioned later. These results are consistent with the S-REF model (16), that negative metacognitive beliefs are closely linked with psychological vulnerability and contribute to stable individual differences marked by personality traits. It is possible that negative metacognitive beliefs can shape how personality is expressed, and it is possible that negative metacognitive beliefs can account for some of the correlations between personality traits and psychological disorder (e.g., 3). However, because the design is cross-sectional and correlational, and because the directional hypothesis tested here was already specified by the S-REF model rather than derived independently from the data, the findings cannot establish that negative metacognitive beliefs cause, underlie, or account for these associations. The reverse direction, for example, trait neuroticism fostering negative metacognitive beliefs, or a shared common cause are alternative explanations that the present data cannot rule out. However, they are not consistent with theory (14) and neuroticism or other personality traits do not provide a parsimonious account of individual differences in metacognitive knowledge, since they describe behavioural and cognitive patterns rather than control functions. The S-REF model suggests that modifying negative metacognitive beliefs, the key target in metacognitive therapy (17), can improve psychological distress (i.e., anxiety and depression) and vulnerability (e.g. neuroticism and other “downstream” traits) simultaneously. However, the present findings do not by themselves establish that modifying negative metacognitive beliefs would improve psychological distress and personality-linked vulnerability. Despite this, it is notable that negative metacognitive beliefs are a treatment target in metacognitive therapy (17) and that a randomized controlled trial of metacognitive therapy versus cognitive-behavioural therapy for generalized anxiety disorder found reductions in both emotional distress and neuroticism (40, 41).

The current study has important limitations that should be acknowledged. Participants were psychology students, predominantly young women, recruited via convenience sampling during a university lecture as part of an introductory psychology course. Due to the substantial total burden of completing the self-report measures (NEO-PI-R and MCQ-30), no information was collected on current psychiatric symptoms, diagnosis, or treatment history, and only limited demographic information was gathered. The findings therefore cannot support conclusions about psychopathological mechanisms or treatment targets in clinical populations, and generalizability beyond similar nonclinical student samples is unknown. Second, the six-node network is low-dimensional (15 possible edges), and graphical LASSO regularization in this setting may introduce bias without a clear inferential advantage. We therefore also report the complete zero-order and unregularized partial-correlation matrices with confidence intervals, and an unregularized GGM sensitivity analysis, in the **Supplementary Material**. Third, the MCQ-30 negative-beliefs subscale assesses beliefs about worry, and although Neuroticism does not assess beliefs about worry, worry and neuroticism are closely connected with emotion. Thus, part of the observed association may therefore reflect similar content or common-method variance rather than a relationship between fully distinct constructs. A full latent-variable (SEM) test of discriminant validity is noted as a priority for future work. Fourth, the present network and DAG include only the MCQ-30 negative-beliefs (uncontrollability-and-danger) subscale. The other four MCQ-30 subscales were not modelled, so the findings speak to this specific facet of metacognition rather than to metacognition or the S-REF model as a whole. Fifth, the directed ordering generated by the DAG rests on strong assumptions, including causal sufficiency, correct model specification, and absence of unmeasured confounding, that cannot be verified in cross-sectional, contemporaneous self-report data. We therefore present the DAG as exploratory throughout and report the completed partial DAG (CPDAG) indicating which arc directions are identifiable from the observed conditional independencies in the **Supplementary Material**. The directional hypothesis tested here was specified in advance by the S-REF model, so recovering it is consistent with, but does not independently test, that model. The reverse direction and a shared-common-cause explanation cannot be ruled out by the present design. Future studies should include more domains of dysfunctional metacognitive belief domains and employ longitudinal or experimental designs to test directional and causal hypotheses about the role of S-REF metacognitions in personality expression.

In conclusion, the present study suggests that negative metacognitive beliefs about the uncontrollability and danger of worry are strongly associated with, and structurally prominent alongside, neuroticism in the network, and may be central to understanding psychological vulnerability marked by specific personality traits such as neuroticism. The implication is that the metacognitive model may have the potential to inform the mechanisms underlying personality features and their association with psychological dysfunction. The exploratory DAG results are consistent with but cannot definitively establish the S-REF model’s prediction that these beliefs may precede and contribute to personality-linked vulnerability. Longitudinal and experimental designs are needed to test this proposed directional hypothesis but the present results support the potential value of examining the contribution of S-REF metacognitions to personality dimensions.

## Data Availability

The raw data supporting the conclusions of this article will be made available by the authors, without undue reservation.
