# Supplementary material for: Partial and directional relationships between negative metacognitive beliefs and the big five personality traits
Source: Front Psychiatry. 2026 Sep 11;17:1897206. doi: 10.3389/fpsyt.2026.1897206 (PMC13612473; doi:10.3389/fpsyt.2026.1897206)
Supplement: Supplementary file 1 [file SupplementaryFile1.docx]

**SUPPLEMENTARY MATERIALS**

**Article Title:**

**Journal Name:**

Corresponding author:

**Descriptive Statistic for Variables in Study 1**

```
 Variable   n   Mean Std.Dev Median    Min    Max   25th   75th   Skew Kurtosis
1      NEG 296 10.274   3.802  9.000  6.000 23.000  7.000 12.000  1.096    3.692
2 NEUROTIC 296 53.015   9.739 52.658 25.547 78.628 46.600 59.471  0.185    2.670
3    EXTRA 296 51.636  10.594 52.521 11.551 74.240 45.117 58.938 -0.626    3.748
4     OPEN 296 54.006   9.120 54.721 23.251 74.841 48.401 60.396 -0.312    2.996
5    AGREE 296 50.555   9.936 51.497 16.946 75.810 44.459 57.415 -0.422    3.355
6    CONSC 296 48.036  10.767 48.288 16.093 70.926 41.689 55.834 -0.357    2.866
```

NEG	Negative beliefs about uncontrollability and danger

NEUROTIC	Neuroticism

EXTRA	Extraversion

OPEN	Openness

AGREE	Agreeableness

CONSC	Consciousness

**Mardia’s multivariate normality test**

Test Statistic	p.value	Method	MVN

1 Mardia Skewness	182.311	<0.001	asymptotic	Not normal

2 Mardia Kurtosis	4.356	<0.001	asymptotic	Not normal

**Centrality values**

```
         Betweenness   Closeness  Strength 	ExpectedInfluence
NEG                0 0.020131114 0.6723952        0.67239518
NEUROTIC           7 0.023589384 0.9843405        0.11419873
EXTRA              3 0.022403358 0.6328658        0.25499944
OPEN               0 0.018321866 0.4484240        0.44842399
AGREE              0 0.009008013 0.1496968        0.05845273
CONSC              0 0.016040932 0.3172394       -0.08379187
```

**Zero-order correlation matrix among the six analytic variables (n = 296).**

|  | **NEG** | **NEUROTIC** | **EXTRA** | **OPEN** | **AGREE** | **CONSC** |
| --- | --- | --- | --- | --- | --- | --- |
| NEG | — | .63 | -.19 | .08 | -.02 | -.03 |
| NEUROTIC |  | — | -.29 | -.05 | -.14 | -.29 |
| EXTRA |  |  | — | .39 | .15 | .17 |
| OPEN |  |  |  | — | .14 | .04 |
| AGREE |  |  |  |  | — | .03 |
| CONSC |  |  |  |  |  | — |

***95% confidence intervals and p-values (pairwise, unadjusted):***

| **Pair** | ***r*** | ***95% CI*** | ***p*** |
| --- | --- | --- | --- |
| NEG–NEUROTIC | .63 | [.56, .70] | < .001 |
| NEG–EXTRA | -.19 | [-.30, -.08] | < .001 |
| NEG–OPEN | .08 | [-.03, .19] | .16 |
| NEG–AGREE | -.02 | [-.14, .09] | .70 |
| NEG–CONSC | -.03 | [-.14, .08] | .60 |
| NEUROTIC–EXTRA | -.29 | [-.39, -.18] | < .001 |
| NEUROTIC–OPEN | -.05 | [-.16, .07] | .43 |
| NEUROTIC–AGREE | -.14 | [-.25, -.03] | .02 |
| NEUROTIC–CONSC | -.29 | [-.40, -.19] | < .001 |
| EXTRA–OPEN | .39 | [.29, .49] | < .001 |
| EXTRA–AGREE | .15 | [.03, .26] | .01 |
| EXTRA–CONSC | .17 | [.06, .28] | < .001 |
| OPEN–AGREE | .14 | [.03, .25] | .02 |
| OPEN–CONSC | .04 | [-.08, .15] | .51 |
| AGREE–CONSC | .03 | [-.09, .14] | .65 |

**Regularized partial-correlation (Adjacency) matrix (EBICglasso, gamma = 0.5), used as input to Figure 1A**

NEG	NEUROTIC	EXTRA	OPEN	AGREE	CONSC

NEG	0.00000000 0.54926961 0.00000000 0.07258543 0.00000000 0.05054014

NEUROTIC	0.54926961 0.00000000 -0.18893317 0.00000000 -0.04562205 -0.20051565

EXTRA	0.00000000 -0.18893317 0.00000000 0.32475637 0.05299259 0.06618364

OPEN	0.07258543 0.00000000 0.32475637 0.00000000 0.05108219 0.00000000

AGREE	0.00000000 -0.04562205 0.05299259 0.05108219 0.00000000 0.00000000

CONSC	0.05054014 -0.20051565 0.06618364 0.00000000 0.00000000 0.00000000

**Unregularized partial-correlation matrix**

Unregularized partial correlations (no LASSO shrinkage), computed by inverting the zero-order correlation matrix and cross-checked with *ppcor::pcor* (*p*-values below). Reported alongside the regularized (glasso) values above for direct comparison.

| **Pair** | **Glasso (regularized)** | **Unregularized partial *r*** | ***p*** |
| --- | --- | --- | --- |
| NEG — NEUROTIC | 0.549 | 0.624 | < .001 |
| NEG — EXTRA | 0.000 | -0.075 | .204 |
| NEG — OPEN | 0.073 | 0.167 | .004 |
| NEG — AGREE | 0.000 | 0.078 | .182 |
| NEG — CONSC | 0.051 | 0.207 | < .001 |
| NEUROTIC — EXTRA | -0.189 | -0.161 | .006 |
| NEUROTIC — OPEN | 0.000 | -0.037 | .532 |
| NEUROTIC — AGREE | -0.046 | -0.136 | .020 |
| NEUROTIC — CONSC | -0.201 | -0.321 | < .001 |
| EXTRA — OPEN | 0.325 | 0.389 | < .001 |
| EXTRA — AGREE | 0.053 | 0.081 | .170 |
| EXTRA — CONSC | 0.066 | 0.119 | .043 |
| OPEN — AGREE | 0.051 | 0.076 | .194 |
| OPEN — CONSC | 0.000 | -0.046 | .437 |
| AGREE — CONSC | 0.000 | -0.076 | .196 |

The regularized and unregularized networks agree closely in sign and relative magnitude. Every pair that *glasso* shrank to exactly zero (NEG–EXTRA/AGREE, NEUROTIC–OPEN, OPEN–CONSC, AGREE–CONSC) was also non-significant in the unregularized matrix (all *p* > .17), suggesting the regularization did not remove any reliable association. The four edges emphasized in the main text (NEG–NEUROTIC, NEUROTIC–EXTRA, NEUROTIC–CONSC, EXTRA–OPEN) are large and significant (*p* < .01) in both the regularized and unregularized matrices.

**Fig. S1.** Expected Influence, Betweenness, and Closeness centrality estimates for the network structure based on standardized scores

**Unregularized GGM plot**

The edge pattern is materially similar to the regularized network

**Fig. S2.** Unregularized GGM plot

**Fig. S3A**. Bootstrapped confidence intervals (CIs) of the edge weights. The red line indicates the edge weight values, and the grey area represents 95% CIs. **Fig. S3B.** Gray boxes indicate edges that do not differ significantly from one another, and black boxes represent edges that differ significantly from one another.

**Fig. S4A**. Average correlation between centrality indices of the network estimation sampled with persons dropped and the original sample. The expected influence permits cautions interpretation. **Figure S4B**. Bootstrapped difference test between node expected influence. Black boxes represent nodes that do differ significantly from one another, and grey boxes indicate nodes that do not differ significantly from one another

**Completed partial DAG / Markov equivalence class**

Reports which arc directions in the DAG are identifiable from the observed conditional independencies (compelled arcs in the CPDAG) versus not identifiable (unoriented). Computed for both the final averaged network (5 arcs) and the single best-fitting DAG (6 arcs, additionally including NEG→OPEN).

| **Arc** | **Compelled / Unoriented** | **Notes** |
| --- | --- | --- |
| NEG → NEUROTIC | Compelled |  |
| CONSC → NEUROTIC | Compelled |  |
| AGREE → NEUROTIC | Compelled |  |
| NEUROTIC → EXTRA | Compelled |  |
| EXTRA → OPEN | Compelled |  |
| NEG → OPEN | Compelled | Present only in the single best-fitting DAG (not the final averaged network); bootstrap arc strength = 0.4988, exactly at the optimal threshold (not retained; see next). |

No arcs were unoriented in either equivalence class

**Full bootstrap arc-strength table**

All 15 node pairs among the 6 nodes, with bootstrap strength (probability the arc is present, in either direction, across the 10,000 bootstrapped networks) and directional probability in each direction. Optimal inclusion threshold for this sample = 0.4988 (Scutari & Nagarajan, 2013); pairs above threshold are retained in the final network.

| **Pair** | **Strength** | **P(A→B)** | **P(B→A)** | **Retained arc** |
| --- | --- | --- | --- | --- |
| NEG — NEUROTIC | 1.000 | .840 (A→B) | .161 | NEG → NEUROTIC |
| EXTRA — OPEN | 1.000 | .503 (A→B) | .497 | EXTRA → OPEN (≈chance) |
| NEUROTIC — CONSC | 0.995 | .154 | .846 (B→A) | CONSC → NEUROTIC |
| NEUROTIC — EXTRA | 0.810 | .861 (A→B) | .139 | NEUROTIC → EXTRA |
| NEUROTIC — AGREE | 0.550 | .292 | .708 (B→A) | AGREE → NEUROTIC |
| NEG — OPEN | 0.499 | .784 (A→B) | .216 | — (at threshold, not retained) |
| EXTRA — CONSC | 0.348 | .327 | .673 | — (below threshold) |
| EXTRA — AGREE | 0.330 | .722 | .278 | — (below threshold) |
| OPEN — AGREE | 0.326 | .616 | .384 | — (below threshold) |
| NEG — EXTRA | 0.220 | .884 | .116 | — (below threshold) |
| NEG — CONSC | 0.186 | .546 | .454 | — (below threshold) |
| NEUROTIC — OPEN | 0.081 | .153 | .847 | — (below threshold) |
| AGREE — CONSC | 0.054 | .492 | .508 | — (below threshold) |
| NEG — AGREE | 0.042 | .287 | .713 | — (below threshold) |
| OPEN — CONSC | 0.016 | .284 | .716 | — (below threshold) |

Algorithm settings: hill-climbing (hc), score = BIC-Gaussian (bic-g); initial fit restart = 50, perturb = 100; bootstrap R = 10,000, algorithm.args = restart 5 / perturb 10 (set.seed(123) before both steps, fully reproducible). NEG–OPEN falls exactly at the optimal threshold (0.4988) and is therefore not retained under the strict “> threshold” rule used by averaged.network(); this boundary case is noted explicitly here for transparency.
